# Variation in IL-21-secreting circulating follicular helper T cells in Kawasaki disease

**DOI:** 10.1186/s12865-018-0282-8

**Published:** 2018-12-27

**Authors:** Meng Xu, Yanfang Jiang, Jian Zhang, Yan Zheng, Deying Liu, Lishuang Guo, Sirui Yang

**Affiliations:** 1 Department of Pediatric Rheumatology and Allergy, The First Hospital of Jilin University, Changchun, 130021 China; 2 Genetic Diagnosis Center, The First Hospital of Jilin University, Changchun, 130021 China; 3 Key Laboratory of Zoonoses Research, Ministry of Education, The First Hospital of Jilin University, Changchun, 130021 China; 4 Jiangsu Co-innovation Center for Prevention and Control of Important Animal Infectious Diseases and Zoonoses, Yangzhou, 225009 China; 5 Department of Pediatric, Children’s Hospital, Changchun, 130021 China; 6 Department of Pediatric Rheumatology and Immunology, Wuhan Children’s Hospital, Tongji Medical College, Huazhong University of Science & Technology, Wuhan, 430000 China

**Keywords:** Circulating follicular helper T cells, Kawasaki disease, Interleukin-21, Immune response

## Abstract

**Objective:**

Circulating follicular helper T (cTfh) cells are a specialized subset of CD4^+^ T cells that express the CXC-chemokine receptor 5 (CXCR5). These cells exhibit immune activities by inducing B cell differentiation and proliferation via the secretion of interleukin (IL)-21. Multiple studies have demonstrated that cTfh cells are associated with the progression and severity of numerous diseases. To investigate the role of cTfh cells in the development of Kawasaki disease (KD), we analyzed the distinct subpopulations of cTfh cells and serum IL-21 levels in different phases of KD.

**Methods:**

According to the differential expression of inducible co-stimulator (ICOS) and programmed cell death protein 1 (PD-1), cTfh cells were divided into distinct subsets. We used flow cytometry and flow cytometric bead arrays (CBA) to analyze subsets of CD4^+^CXCR5^+^ T cells and serum IL-21 levels. The samples were collected from control subjects and Kawasaki disease patients in the acute and remission phases.

**Results:**

In the acute phase (AP), the percentages of ICOS^high^PD-1^high^, ICOS^+^PD-1^+^, ICOS^−^PD-1^+^, CD45RA^−^IL-21^+^ cTfh cells and serum IL-21 levels significantly increased. Furthermore, the percentages of ICOS^high^PD-1^high^ and ICOS^+^PD-1^+^ cTfh cells positively correlated with erythrocyte sedimentation rate (ESR) and C-reactive protein (CRP) values, whereas the percentage of ICOS^−^PD-1^+^ cTfh cells indicated negative correlations. The percentages of ICOS^+^PD-1^+^, ICOS^high^PD-1^high^ and CD45RA^−^IL-21^+^ cTfh cells correlated positively with serum IL-21 levels. In the remission phase (RP), the percentages of ICOS^−^PD-1^+^, CD45RA^−^IL-21^+^ cTfh cells and serum IL-21 levels were significantly decreased. In contrast, the percentages of ICOS^+^PD-1^+^, ICOS^high^PD-1^high^, and ICOS^+^PD-1^−^ cTfh cells were further increased. Among these subsets, only CD45RA^−^IL-21^+^ cTfh cells correlated positively with serum IL-21 levels.

**Conclusions:**

The present study is the first investigation that examined the distribution of circulating cTfh cell subsets in Kawasaki disease. Both cTfh cells and serum IL-21 are essential to the pathogenesis of KD. Our study provides further understanding of the immune response involved in KD and offers novel insights in the pathogenetic mechanism of this disease.

**Electronic supplementary material:**

The online version of this article (10.1186/s12865-018-0282-8) contains supplementary material, which is available to authorized users.

## Background

Kawasaki disease (KD) is an acute febrile vasculitis that was first reported in 1967 [1]. KD most commonly occurs in infants and children under five years of age and is characterized initially by high fever, mucocutaneous inflammation, edema of the extremities, polymorphous rash, and cervical lymphadenopathy (≥1.5 cm diameter) [2]. The exact pathogenesis of Kawasaki disease remains unknown. Generally, it is believed that KD is secondary to infectious agents, triggering an aberrant immune response in genetically susceptible individuals [3, 4]. The treatment efficacy of intravenous immunoglobulin (IVIG) administered during the acute phase of the disease further confirms the fundamental involvement of immune response dysregulation in the pathogenesis of KD. Therefore, the understanding of the factors that regulate the abnormal immune response in KD is important for developing strategies for the management of KD patients.

Both innate and adaptive immune systems are involved in the pathogenesis of KD. The innate immune system presents high numbers of activated circulating neutrophils and elevated levels of serum cytokines, such as interleukin (IL)-1, IL-6, and tumor necrosis factor alpha (TNFα) [5]. The reduction in the numbers of neutrophils and cytokines levels following IVIG administration [6] is regarded as a consequence of the downregulation of the nuclear factor kappa B (NF-κB) signaling pathway in monocytes/macrophages [7]. Although T cells have traditionally been considered to play essential roles in adaptive immune responses, the alterations in the percentage of T cells in KD are a subject of controversy and different investigators have reported conflicting results [8–10]. It has been concluded that the variation in T cells could be further investigated based on of their subpopulations and cytokine levels [5]. Current studies that examined the variation of T-cell subpopulations with regard to KD have generally investigated T helper cells [11, 12], regulatory T cells [13], and cytotoxic T cells [14]; however, the role of follicular helper T (Tfh) cells in KD remains to be elucidated. B cells are also important participants in the adaptive immune responses. In acute phase of KD, studies about B cells found increased CD19^+^ cells [15] and elevated levels of serum immunoglobulins (IgA, IgG, IgM and IgE) [16]. Accordingly, it can be speculated that Tfh cells may be involved in the pathogenesis of KD due to their ability in homing B cell to the germinal centers (GC), regulating GC positive selection, and inducing B cell differentiation to plasma cells and memory B cells via the secretion of IL-21 [17–19]. However, suppressed plasmablast responses [20] and reduced IgA-expressing B cells [21] in KD were also reported. Hence, the studies conducted on Tfh cells are important in clarifying the role of B cells in KD, since Tfh cells can aid the function of B cells.

Tfh cells were initially identified in the GC of secondary lymphoid tissues and have been shown to express the CXC-chemokine receptor 5 (CXCR5). The percentage of Tfh cells can be detected by the expression of inducible co-stimulator (ICOS) and programmed cell death protein 1 (PD-1), two CD28 superfamily molecules [22, 23]. ICOS delivers positive signals to CD4^+^ T cells by interacting with dendritic cells and B cells that express an ICOS ligand, which is involved in Tfh cell differentiation [24, 25]. In contrast to ICOS, the inhibitory signals [26, 27] that block the interactions between PD-1 and the ligands PD-L1 and/or PD-L2 have been shown to increase the differentiation of Tfh cells [28, 29]. The assessment of GC Tfh cells in patients, particularly in children, is subject to huge limitations, due to poor access to lymphoid organ samples. Despite these disadvantages, the establishment of circulating Tfh (cTfh) cells [26] from blood samples offers a surrogate strategy to analyze Tfh responses. Circulating Tfh cells are believed to represent a memory compartment of GC Tfh cells that express B-cell lymphoma 6 protein (Bcl-6), which is downregulated when the cells enter the circulation [30]. Circulating Tfh cells share the phenotype and functional properties of GC Tfh cells [31, 32]. Current understanding of cTfh cells suggests that they can be classified into distinct subsets according to the differential expression of ICOS and PD-1. Typically, they were divided into one activated subset (ICOS^+^PD1^++^) and two quiescent subsets (ICOS^−^PD1^−^ and ICOS^−^PD1^+^) [33]. The majority of ICOS^+^PD1^++^ cTfh cells express Ki67, which is a cellular marker for proliferation. On the contrary, the two quiescent subsets lack the expression of Ki67 [34]. Furthermore, in comparison, ICOS^+^PD1^++^ cTfh cells display the most efficient capacity in helping naïve or memory B cells. Thus, investigation on the variation in cTfh-cell subsets would be beneficial for better understanding the level of their activation. IL-21 is recognized as a hallmark for cTfh cells [33] and belongs to the type I cytokine family. This cytokine is a highly potent stimulator of plasma cell proliferation and differentiation [35]. IL-21 is also believed to be an autocrine growth factor for Tfh cells [36]. However, the role of IL-21 in cTfh cells remains to be fully elucidated.

T cells comprise a heterogeneous population; therefore, in the present study, we aimed to analyze the distinct subsets of circulating Tfh cells that were defined by characteristic Tfh markers and serum IL-21 levels. The examination of their expression levels and the correlation among the subgroups can be used to further clarify the immunopathogenesis of KD.

## Materials and methods

### Patients

A total of 24 hospitalized pediatric patients with KD ranging from six months to five years in age and 20 age-matched control subjects were enrolled from May 2015 to November 2016 (14 cases in the Department of Pediatrics, The First Hospital of Jilin University, China and another 10 cases in the Department of Pediatrics, Children’s Hospital, Changchun, China). Among the KD patients, only one was diagnosed with coronary artery lesions by echocardiography. All KD patients underwent detailed physical and laboratory examinations, and were diagnosed according to the criteria of the 2004 American Heart Association (AHA) statement [37]. Patients with other autoimmune diseases were excluded. All individuals were treated with IVIG at a cumulative dose of 2 g/kg within two days and 30–100 mg/kg/d of aspirin in divided doses from the time at which the diagnosis was established until defervescence. Disease remission was achieved following treatment without recurrence in all patients. None of these children had received other medical therapy for at least one month. The samples were collected from patients in both the acute and remission phases of KD. Acute phase (AP) refers to the period from diagnosis establishment to IVIG administration. Remission phase (RP) is defined as the time from which patients are afebrile for at least 48 h before discharge. The control group comprised eight children with hexadactyly, four children with wryneck, and eight children with hernias and without any other autoimmune diseases or infections in the previous month. The samples were obtained before surgery. The study protocol was approved by the ethics committee of The First Hospital of Jilin University. Written informed consent was obtained from the parents of each child.

The following laboratory parameters were recorded: white blood cell count, absolute neutrophil and lymphocyte counts, serum immunoglobulins (Ig) G, A and M, C-reactive protein (CRP) and erythrocyte sedimentation rate (ESR).

### Flow cytometric analysis

Blood samples were collected separately from controls and KD patients in different phases. Peripheral blood mononuclear cells (PBMCs) were isolated from individual KD patients and control subjects by density-gradient centrifugation at 800×*g* for 30 min at 25 °C using Ficoll-Paque Plus (Amersham Biosciences, Little Chalfont, UK). Freshly isolated PBMCs (4 × 10^6^/mL) were cultured in 10% fetal calf serum RPMI-1640 (Hyclone, Logan, UT, USA) in U-bottom 24-well tissue culture plates (Costar, Lowell, MA, USA) and stimulated for 1 h with or without 50 ng/mL of phorbol myristate acetate (PMA) in the presence of 2 μg/mL of ionomycin (Sigma, St. Louis, MO, USA). The cells were then treated with Brefeldin A (10 μg/mL, GolgiStop™, BD Biosciences, San Jose, CA, USA) for an additional 5 h. For flow cytometric analysis, PBMCs were stained in duplicate with BV510-anti-CD3, APC-H7-anti-CD4, BB515-anti-CXCR5, PE-Cy5-anti-CD45RA, PE-CF594-anti-CD279 and BV421-anti-CD278 (Becton Dickinson, San Jose, CA, USA) at room temperature for 30 min. Subsequently, the cells were fixed, permeabilized, and stained with PE-anti-IL-21 (Becton Dickinson). Multicolor flow cytometry (FACSAria™ II, BD Biosciences) was used to determine the percentages of distinct cTfh cells, and the data were analyzed with FlowJo software (v5.7.2; FlowJo, Ashland, OR, USA).

### Measurement of serum IL-21 levels by cytometric bead array (CBA)

Serum IL-21 concentrations were detected using a CBA human soluble protein master buffer kit (BD Biosciences) according to the manufacturer’s instructions. The samples were further analyzed with a flow cytometer (FACSAria™ II, BD Biosciences), and quantified using the CellQuest Pro and CBA softwares (Becton Dickinson).

### Statistical analysis

Statistical data were performed with SPSS version 22.0 software. A *P* value lower than 0.05 (*P* < 0.05) was considered to indicate statistical significance. All data were expressed as the median and range values. The differences between groups were analyzed using the Kruskal–Wallis test. The Wilcoxon matched pairs test was applied to assess the differences between the AP and RP data. The relationship between different variables was evaluated using the Spearman’s rank correlation test.

## Results

### Patient characteristics

A total of 24 patients with KD and 20 control subjects were recruited in the present study. Five samples of KD patients in RP were unavailable due to advanced discharge. The demographic and clinical characteristics of patients and control subjects are shown in Table 1. No significant differences were detected with regard to the paremeters age and sex between the KD and the control groups. Although white blood cell count and the number of absolute neutrophils were significantly higher in the KD group, no significant differences were noted in absolute lymphocyte counts between the two groups. The CRP and ESR values were significantly elevated in the KD group compared with that noted in the control group. In addition, serum IgG levels were significantly lower in the KD group than those in the control group. However, no significant differences were noted in the serum IgM and IgA levels between the two groups. In the RP, the white blood cell count, absolute neutrophil count and CRP values were dramatically decreased compared with those in the AP.

**Table 1:** The demographic and clinical characteristics of the study participants

Parameters	Kawasaki disease		Controls (*n* = 20)
	Acute phase (*n* = 24)	Remission phase (*n* = 19)	
Age, year	2.75 (0.6–5)	NA	2.38 (0.9–4.5)
Sex, Female/Male	11/14	NA	8/12
CRP, mg/L	76.25 (20–151)^#, ∗^	2.76 (0.8–5.28)	2.35 (0.7–3.28)
ESR, mm/h	51.37 (11–98)^#^	53.67 (9–76)^#^	7 (3–11)
IgG, g/L	6.295 (1.82–16.1)^#^	NA	10.22 (6.08–14.37)
IgA, g/L	1.715 (0.91–2.92)	NA	1.91 (0.71–3.1)
IgM, g/L	0.99 (0.19–1.77)	NA	1.035 (0.69–1.93)
WBC, 10^9^/L	14.31 (5.89–35)^#, ∗^	8.729 (4.82–13.79)	7.735 (5.31–10.03)
Neutrophils, 10^9^/L	10.14 (2.52–33.69)^#, ∗^	2.98 (1.98–5.09)	2.58 (2.06–4.69)
Lymphocytes, 10^9^/L	2.915 (0.88–8.82)	3.736 (1.92–6.75)	4.085 (1.01–5.28)

Data shown are median (range) or number of cases. *CRP* C-reactive protein, *ESR* erythrocyte sedimentation rate, *Ig* immunoglobulin, *WBC* white blood cell counts, *NA* not available. ^#^*P* < 0.05 vs. the controls. ^*^P < 0.05 vs. remission phase

### Circulating CD4^+^CXCR5^+^ T cells subsets and serum IL-21 levels in the different phases of KD

**Fig. 1:**
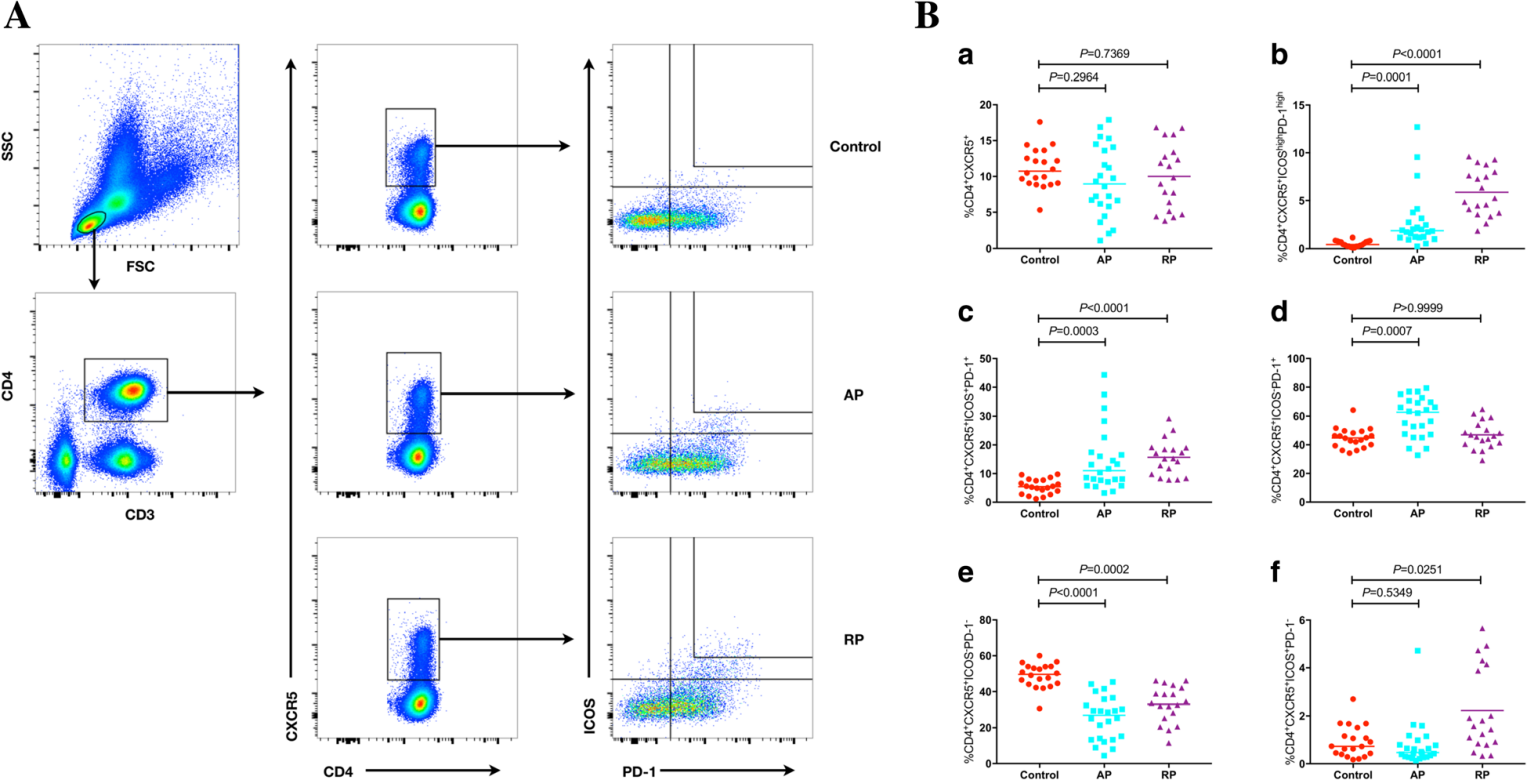
Flow cytometry analysis of the frequency of circulating Tfh cells in KD patients. **A** Flow cytometry analysis. **B** Quantitative analysis. The data shown are representative dot plots or are expressed as the mean percentage of T cells of individual subjects. The horizontal lines represent the median values. PBMC, peripheral blood mononuclear cell. AP, Acute phase. RP, Remission phase

**Fig. 2:**
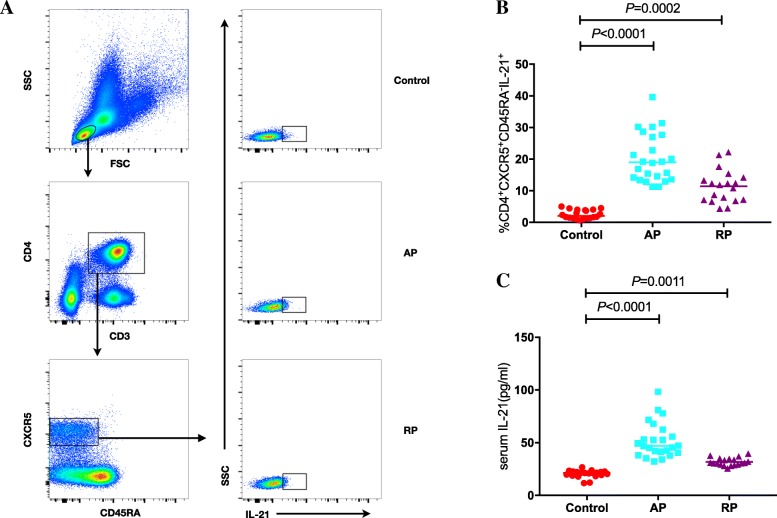
Flow cytometry analysis of the frequency of circulating Tfh cells in KD patients. **a** Flow cytometry analysis. **b** Quantitative analysis of CXCR5 + CD45RA- IL-21+ T cells. **c** Quantitative analysis of serum IL-21 concentration

To investigate the role of circulating Tfh cells in KD, PBMCs from control subjects and patients in different phases of KD were immunostained for CD3, CD4, CXCR5, CD278, CD279, CD45RA and IL-21 expression, and further analyzed by flow cytometry. Initially, five subsets of cTfh cells were described by flow cytometry that were based on the differential expression of ICOS and PD-1, namely CD4^+^CXCR5^+^ICOS^high^PD-1^high^, CD4^+^CXCR5^+^ICOS^+^PD-1^+^, CD4^+^CXCR5^+^ICOS^−^PD-1^+^, CD4^+^CXCR5^+^ICOS^−^PD-1^−^ and CD4^+^CXCR5^+^ICOS^+^PD-1^−^. To ensure proper gating strategy, isotype controls were used to determine the gating parameters (Additional file 1: Figure S1). These cell populations were measured by gating initially live lymphocytes, subsequently CD3^+^CD4^+^ T cells, and finally CD4^+^CXCR5^+^ T cells (Fig. 1A). CD4^+^CXCR5^+^ T cells were considered circulating Tfh cells. No significant differences were noted in the percentages of total cTfh cells in the AP and RP KD groups compared with the control group (*P* = 0.2964 and *P* = 0.7369, respectively; Fig. 1Ba). The percentages of ICOS^high^PD-1^high^ cells were significantly higher in both the AP and RP groups than those noted in the control groups (*P* = 0.0001 and *P* < 0.0001, respectively; Fig. 1Bb) and a similar pattern was observed in the percentages of ICOS^+^PD-1^+^ cells (*P* = 0.0003 and *P* < 0.0001, respectively; Fig. 1Bc). However, the percentage of ICOS^−^PD-1^+^ cells was significantly increased in the AP, but not in the RP groups (*P* = 0.0007 and *P* > 0.9999, respectively; Fig. 1Bd). In contrast, the percentages of ICOS^−^PD-1^−^ cTfh cells in both the AP and RP were significantly lower than those in the control group (*P* < 0.0001 and *P* = 0.0002, respectively; Fig. 1Be). Moreover, a significant elevation in the percentage of ICOS^+^PD-1 cTfh cells was noted only in the RP of KD and not in the AP (*P* = 0.0251 and *P* = 0.5349, respectively; Fig. 1Bf). Subsequenlty, we analyzed the serum IL-21 levels and the percentage of the T cell subpopulation, namely CD4^+^CXCR5^+^CD45RA^−^IL-21^+^ T cells, by gating initially live lymphocytes, subsequently CD3^+^CD4^+^ T cells, and finally CXCR5^+^CD45RA^−^ T cells (Fig. 2a). A significant increase in the percentage of CD45RA^−^IL-21^+^ cTfh cells was observed in both AP and RP KD patients (*P* < 0.0001 and *P* = 0.0002, respectively; Fig. 2b). Similarly, serum IL-21 levels were significantly elevated in both the AP and RP (*P* < 0.0001 and *P* = 0.0011, respectively; Fig. 2c). The data indicated that although the overall percentage of cTfh cells remained stable, significant variations were noted among the cTfh cell subpopulations and serum IL-21 levels. This further suggested that the RP represents a transitional period from the AP to recovery, in which clinical symptoms and characteristics improve, despite the continuation of the immune response.

### Correlation of the percentages of distinct cTfh-cells subsets with clinical characteristics and IL-21 levels in the AP of KD

**Fig. 3:**
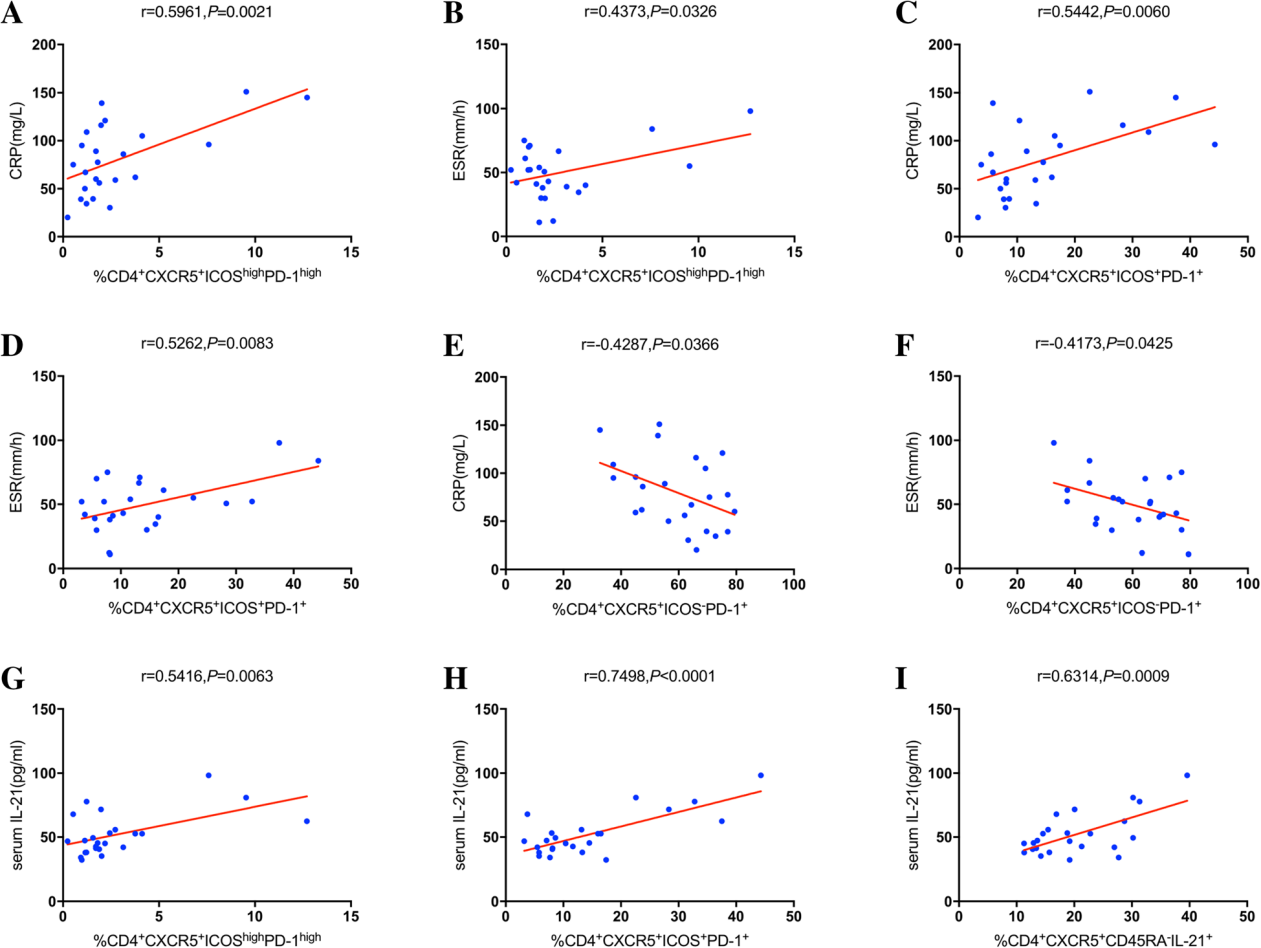
Correlation analysis during the acute phase of KD. **a–h** The correlation between the subsets of cTfh cells and the serum CRP and IL-21 levels and/or ESR

To understand the importance of cTfh cells in the pathogenesis of KD, the associations of the percentages of distinct cTfh-cells subsets with the levels of the biomarkers CRP and ESR and of the IL-21 in the AP were analyzed. We demonstrated that both serum CRP and ESR levels positively correlated with the percentage of ICOS^high^PD-1^high^ cTfh cells (*r* = 0.5961, *P* = 0.0021, Fig. 3a; *r* = 0.4373, *P* = 0.0326, respectively; Fig. 3b). A similar pattern was observed for the percentage of ICOS^+^PD-1^+^ cTfh cells (*r* = 0.5442, *P* = 0.0060, Fig. 3c; *r* = 0.5262, *P* = 0.0083, respectively; Fig. 3d). In contrast to this finding, serum CRP levels and serum ESR negatively correlated with the percentage of ICOS^−^PD-1^+^ cTfh cells (*r* = 0.4287, *P* = 0.0366, Fig. 3e; *r* = 0.4173, *P* = 0.0425, respectively; Fig. 3f). Furthermore, serum IL-21 levels positively correlated with the percentage of ICOS^high^PD-1^high^ (*r* = 0.5416, *P* = 0.0063, Fig. 3g), ICOS^+^PD-1^+^ (*r* = 0.7498, *P* < 0.0001, Fig. 3h) and CD45RA^−^IL-21^+^ (*r* = 0.6314, *P* = 0.0009, Fig. 3i) cTfh cells. No additional significant correlations were noted among these parameters (data not shown). The data indicated that different cTfh-cell subsets play distinct roles in acute KD.

### Variation of cTfh-cells subsets and serum IL-21 levels after treatment

**Fig. 4:**
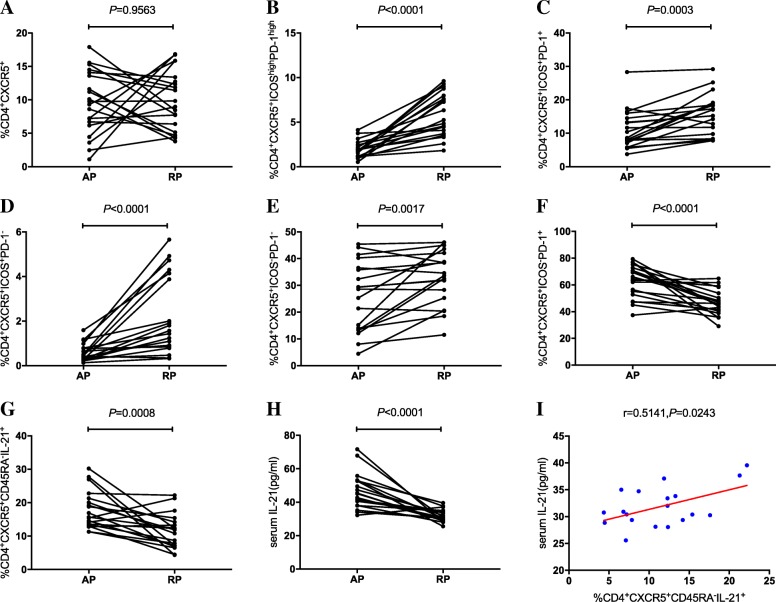
**a–h** The variations of cTfh-cells subsets and serum IL-21 levels in the remission phase. **i** Correlations between subsets of cTfh cells and the levels of serum IL-21

To further investigate the role of cTfh cells in KD patients, we explored the percentages of distinct cTfh-cells subsets in the RP. As shown in Fig. 4, heterogeneous variations were noted in the cTfh-cell subsets, although this effect was not statistically significant in the CD4^+^CXCR5^+^ T cells (*P* = 0.9563, Fig. 4a). However, the percentages of ICOS^high^PD-1^high^ and ICOS^+^PD-1^+^ cTfh cells further increased in the RP (*P* < 0.0001, Fig. 4b; *P* = 0.0003, Fig. 4c). Furthermore, the percentages of ICOS^+^PD-1^−^ and ICOS^−^PD-1^−^ cTfh cells were also significantly increased (*P* < 0.0001, Fig. 4d; *P* = 0.0017, Fig. 4e). In contrast to these observations, the percentages of ICOS^−^PD-1^+^ cTfh cells and CD45RA^−^IL21^+^ cTfh cells, as well as the serum IL-21 levels were significantly decreased (*P* < 0.0001, Fig. 4f; *P* = 0.0008, Fig. 4g; *P* < 0.0001, Fig. 4h). The data of the present study demonstrated that certain cTfh cell subsets further increased following drug administration and clinical improvement. It was notable that in the RP, only the CD45RA^−^IL21^+^ cTfh cells correlated with serum IL-21 levels (*r* = 0.5141, *P* = 0.0243, Fig. 4i).

## Discussion

The roles of circulating Tfh cells in different disease models have already been widely investigated. A previous study conducted on systemic lupus erythematosus indicated that PD-1 expression on cTfh cells contributed to the regulation of germinal center B-cell function and humoral response [38]. Szabo et al. reported that ICOS^+^PD-1^+^ cTfh cells and IL-21-producing CD4^+^CXCR5^+^PD-1^+^ T cells were significantly increased in Sjögren’s syndrome subjects [39]. Our previous study in children indicated that the percentage of cTfh cells correlated with the pathogenesis and progression of Henoch–Schönlein purpura [40]. In the present study, we found that although the overall percentage of cTfh cells remained stable, marked variations were noted in the percentages of cTfh-cell subsets and the levels of cytokines. In the AP, the percentages of ICOS^high^PD-1^high^, ICOS^+^PD-1^+^, ICOS^−^PD-1^+^, and CD45RA^−^IL-21^+^ cTfh cells and serum IL-21 levels were significantly elevated. It is apparent that aberrant distribution of cTfh cells is ubiquitous in rheumatic diseases, although the precise mechanism remains unclear. Based on our data, ICOS^high^PD-1^high^, ICOS^+^PD-1^+^, ICOS^−^PD-1^+^ cTfh cells correlated positively with CRP and ESR, which suggested that these three subsets contribute to innate immune responses. Correlation analysis with IL-21 levels implied that ICOS^high^PD-1^high^, ICOS^+^PD-1^+^ and CD45RA^−^IL-21^+^ cTfh cells affected KD development via IL-21 secretion. It seems difficult to explain that although the clinical symptoms had been improved in the RP, the percentages of the ICOS^high^PD-1^high^ and ICOS^+^PD-1^+^, and ICOS^+^PD-1^−^ cTfh cells were further increased. However, among these subsets, ICOS^high^PD-1^high^ population may be a representative subset of cTfh cells. Not only because ICOS^high^PD-1^high^ population can be regarded as a subpopulation of ICOS^+^PD1^++^ cTfh cells, which is an activated subset, but also because this phenotype is similar to that of GC Tfh cells, which are ICOS^++^PD-1^++^ [35]. In other words, the increase of ICOS^high^PD-1^high^ cTfh cells likely represents the activation of cTfh cells. Hence, our results suggested that cTfh cells were activated in the AP, and they were persistently activated in the RP. Although the function of these subsets cannot be clarified in the present analysis, the absence of a correlation with IL-21 levels despite the increased percentages of these subsets, indicates that these cells may not secrete IL-21 efficiently in RP [34, 41, 42]. Furthermore, Franco et al. [43] indicated that the expansion of circulating central memory T cells but not effector memory T cells could be detected even at 1 to 3 months following the onset of the disease. This expansion aims to protect patients against future exposure to the stimuli of KD. Whether these further increased subsets in RP are circulating central memory cells remains to be clarified.

Since its discovery in 2000, IL-21 has been shown to perform antitumor and antiviral functions and to participate in the development of autoimmune diseases via the Janus kinase (JAK)-signal transducer, the activator of transcription (STAT), the mitogen-activated protein kinase (MAPK) and the phosphoinositide 3-kinase (PI3K)-AKT signaling pathways [44]; however, the mechanism underlying the role in KD is unknown. Several studies have been conducted on the role of IL-21 in KD. One study in a Korean cohort demonstrated that IL-21 represents a sensitive and specific biomarker of KD [45]. In accordance with this report, we found elevated serum IL-21 levels in the AP, while the corresponding levels were reduced in the RP. However, Engelberg et al. reported that IL-21 levels were also elevated in febrile controls [46]. Therefore, serum IL-21 levels appear to be a sensitive, but not specific, indicator of active KD. Additionally, serum IL-21 levels may be regulated by IgG levels [45]. In the present study, we observed lower levels of serum IgG and higher expression levels of IL-21 in the AP, while in the RP the IgG levels were shown to increase [47] and IL-21 levels decreased. Nevertheless, further analysis indicated no correlation between serum levels of IL-21 and IgG in the AP. It can be speculated that this finding may have been produced by the increase in the normal range of IgG with age. Furthermore, IL-21 is not exclusively expressed in cTfh cells, but also in Th17 and NKT cells [48, 49]. IL-21 was further found to be suppressed by activated plasma cells [50]. Thus, in addition to cTfh cells, multiple factors, such as Th17, NKT and plasma cells, might affect serum IL-21 levels.

The relationship between IL-21 expression and Tfh cell differentiation remains controversial. It has been postulated that IL-21 contributes to the efficient development of Tfh cells by upregulating BCL-6 and MAF [51, 52], while other studies have indicated that Tfh cells can be generated without IL-21 expression [53]. Alternatively, it has been shown that IL-21 alone is insufficient to enhance the expression of CXCR5 [54]. Thus, it can be hypothesized that IL-21 acts as a cooperator, rather than a conductor of Tfh cell differentiation, which is a complex process regulated by IL-21, IL-12, IL-6, BCL-6, and ICOS [35, 54]. In the present study, we investigated the percentage of total cTfh cells and the serum IL-21 levels in KD patients during different phases of the disease progression. It is interesting to note, that no significant variation was noted in the percentage of CXCR5 cells regardless of the levels of IL-21, indicating that no definite correlation between IL-21 levels and cTfh cell differentiation in KD.

Unfortunately, the correlations between coronary artery lesions and cTfh cells were not analyzed in the present study. However, a recent study revealed associations of coronary artery lesions with a number of clinical characteristic, particularly CRP and ESR [55]. It was noted that KD patients with CRP levels higher than 30 mg/L and ESR higher than 40 mm/h exhibited a higher incidence of coronary artery lesion. Moreover, the percentage of ICOS^−^PD-1^+^ cTfh cells correlated negatively with ESR and CRP, indicating the possibility of an increased percentage of ICOS^−^PD-1^+^cTfh cells to act as a protective barrier against coronary artery lesions. This hypothesis may provide a new therapeutic approach in preventing coronary artery lesions. According to our clinical observations, coronary artery lesions were present in the patients without significant elevation of CRP and ESR. In addition, not all of the patients with high CRP and ESR possessed coronary artery lesions. It can be speculated that the pathogenesis of coronary artery lesions in KD is diverse, possibly due to epigenetic effects [56] and genetic polymorphisms [57]. Therefore, investigation of the association between cTfh cells and the development of coronary lesions are required. Besides, another limitation of the present study shoud be mentioned. The exact function and mechanism underlying the further increase in the cTfh cell population during the RP remain to be elucidated. Focus will be given on these issues in the subsequent studies.

## Conclusions

In summary, significant variations were identified in the percentages of cTfh-cell subsets and the level of serum IL-21 in Kawasaki disease. Our results indicated that IL-21-secreting cTfh cells were essential for both the acute and remission phase of KD. It also can be concluded that cTfh cells were activated in the acute phase and persistently activated in remission phase. To the best of our knowledge, this is the first investigation of serum IL-21 levels in terms of the distribution of circulating cTfh cell subsets in KD. Our data provide further understanding of the immune responses of KD and novel insights in the pathogenesis of this disease.

## Additional file

Additional file 1: **Figure S1.** To ensure proper gating stratery, isotype controls were used to determin the gating parameters. (PDF 104 kb)

## Abbreviations

AP: Acute phase
CBA: Cytometric bead array
cTfh cells: Circulating follicular helper T cells
CXCR5: CXC-chemokine receptor 5
GC: Germinal center
ICOS: Inducible co-stimulator
IL: Interleukin
KD: Kawasaki disease
NF-κB: Nuclear factor kappa B
PBMC: Peripheral blood mononuclear cells
PD-1: Programmed cell death protein 1
RP: Remission phase

## References

[1] Kawasaki T (1967). Acute febrile mucocutaneous syndrome with lymphoid involvement with specific desquamation of the fingers and toes in children. Arerugi.

[2] McCrindle B (2017). Diagnosis, treatment, and long-term Management of Kawasaki Disease: a scientific statement for health professionals from the American Heart Association. Circulation.

[3] Shulman ST, Rowley AH (2015). Kawasaki disease: insights into pathogenesis and approaches to treatment. Nat Rev Rheumatol.

[4] Onouchi Y (2012). A genome-wide association study identifies three new risk loci for Kawasaki disease. Nat Genet.

[5] Matsubara T, Ichiyama T, Furukawa S (2005). Immunological profile of peripheral blood lymphocytes and monocytes/macrophages in Kawasaki disease. Clin Exp Immunol.

[6] Furukawa S (1990). Reduction of peripheral blood macrophages/monocytes in Kawasaki disease by intravenous gammaglobulin. Eur J Pediatr.

[7] Ichiyama T (2001). NF-kappaB activation in peripheral blood monocytes/macrophages and T cells during acute Kawasaki disease. Clin Immunol.

[8] Abe J (1992). Selective expansion of T cells expressing T-cell receptor variable regions V beta 2 and V beta 8 in Kawasaki disease. Proc Natl Acad Sci U S A.

[9] Brogan P (2003). T cell Vbeta repertoires in childhood vasculitides. Clin Exp Immunol.

[10] Nomura Y (1998). Twenty-five types of T-cell receptor Vbeta family repertoire in patients with Kawasaki syndrome. Eur J Pediatr.

[11] Kimura J (2004). Th1 and Th2 cytokine production is suppressed at the level of transcriptional regulation in Kawasaki disease. Clin Exp Immunol.

[12] Guo MM (2015). Th17- and Treg-related cytokine and mRNA expression are associated with acute and resolving Kawasaki disease. Allergy.

[13] Ni F (2014). Regulatory T cell microRNA expression changes in children with acute Kawasaki disease. Clin Exp Immunol.

[14] Ye Q (2016). Intravenous immunoglobulin treatment responsiveness depends on the degree of CD8+ T cell activation in Kawasaki disease. Clin Immunol.

[15] Ding Y (2015). Profiles of responses of immunological factors to different subtypes of Kawasaki disease. BMC Musculoskelet Disord.

[16] Lin CY, Hwang B (1987). Serial immunologic studies in patients with mucocutaneous lymph node syndrome (Kawasaki disease). Ann Allergy.

[17] Breitfeld D (2000). Follicular B helper T cells express CXC chemokine receptor 5, localize to B cell follicles, and support immunoglobulin production. J Exp Med.

[18] Schaerli P (2000). CXC chemokine receptor 5 expression defines follicular homing T cells with B cell helper function. J Exp Med.

[19] Shulman Z (2013). T follicular helper cell dynamics in germinal centers. Science.

[20] Martin M, Wrotniak BH, Hicar M (2018). Suppressed plasmablast responses in febrile infants, including children with Kawasaki disease. PLoS One.

[21] Shingadia D (2001). Surface and cytoplasmic immunoglobulin expression in circulating B-lymphocytes in acute Kawasaki disease. Pediatr Res.

[22] Hutloff A (1999). ICOS is an inducible T-cell co-stimulator structurally and functionally related to CD28. Nature.

[23] Sharpe A, Freeman G (2002). The B7-CD28 superfamily. Nat Rev Immunol.

[24] Bossaller L (2006). ICOS deficiency is associated with a severe reduction of CXCR5+CD4 germinal center Th cells. J Immunol.

[25] Nurieva R (2008). Generation of T follicular helper cells is mediated by interleukin-21 but independent of T helper 1, 2, or 17 cell lineages. Immunity.

[26] Crotty S (2014). T follicular helper cell differentiation, function, and roles in disease. Immunity.

[27] Ueno H, Banchereau J, Vinuesa CG (2015). Pathophysiology of T follicular helper cells in humans and mice. Nat Immunol.

[28] Good-Jacobson KL (2010). PD-1 regulates germinal center B cell survival and the formation and affinity of long-lived plasma cells. Nat Immunol.

[29] Hamel KM (2010). B7-H1 expression on non-B and non-T cells promotes distinct effects on T- and B-cell responses in autoimmune arthritis. Eur J Immunol.

[30] Schmitt N, Bentebibel S, Ueno H (2014). Phenotype and functions of memory Tfh cells in human blood. Trends Immunol.

[31] Morita R (2011). Human blood CXCR5(+)CD4(+) T cells are counterparts of T follicular cells and contain specific subsets that differentially support antibody secretion. Immunity.

[32] Ueno H (2016). T follicular helper cells in human autoimmunity. Curr Opin Immunol.

[33] Ueno H, Human Circulating T (2016). Follicular helper cell subsets in health and disease. J Clin Immunol.

[34] Locci M (2013). Human circulating PD-1+CXCR3-CXCR5+ memory Tfh cells are highly functional and correlate with broadly neutralizing HIV antibody responses. Immunity.

[35] Crotty S (2011). Follicular helper CD4 T cells (TFH). Annu Rev Immunol.

[36] King C, Tangye SG, Mackay CR (2008). T follicular helper (TFH) cells in normal and dysregulated immune responses. Annu Rev Immunol.

[37] Newburger JW (2004). Diagnosis, treatment, and long-term management of Kawasaki disease: a statement for health professionals from the committee on rheumatic fever, endocarditis and Kawasaki disease, council on cardiovascular disease in the young, American Heart Association. Circulation.

[38] Choi JY (2015). Circulating follicular helper-like T cells in systemic lupus erythematosus: association with disease activity. Arthritis Rheumatol.

[39] Szabo K (2016). A comprehensive investigation on the distribution of circulating follicular T helper cells and B cell subsets in primary Sjogren's syndrome and systemic lupus erythematosus. Clin Exp Immunol.

[40] Liu D (2016). Distinct phenotypic subpopulations of circulating CD4+CXCR5+ follicular helper T cells in children with active IgA vasculitis. BMC Immunol.

[41] Bentebibel S (2013). Induction of ICOS+CXCR3+CXCR5+ TH cells correlates with antibody responses to influenza vaccination. Sci Transl Med.

[42] Boswell K (2014). Loss of circulating CD4 T cells with B cell helper function during chronic HIV infection. PLoS Pathog.

[43] Franco A (2010). Memory T-cells and characterization of peripheral T-cell clones in acute Kawasaki disease. Autoimmunity.

[44] Spolski R, Leonard W (2014). Interleukin-21: a double-edged sword with therapeutic potential. Nat Rev Drug Discov.

[45] Bae Y (2012). Elevated serum levels of IL-21 in Kawasaki disease. Allergy Asthma Immunol Res.

[46] Engelberg R (2017). Observational study of Interleukin-21 (IL-21) does not distinguish Kawasaki disease from other causes of fever in children. Pediatr Rheumatol Online J.

[47] Han J (2017). Correlation between elevated platelet count and immunoglobulin levels in the early convalescent stage of Kawasaki disease. Medicine (Baltimore).

[48] Coquet J (2013). IL-21 modulates activation of NKT cells in patients with stage IV malignant melanoma. Clin Transl Immunology.

[49] Korn T (2007). IL-21 initiates an alternative pathway to induce proinflammatory T(H)17 cells. Nature.

[50] Pelletier N (2010). Plasma cells negatively regulate the follicular helper T cell program. Nat Immunol.

[51] Bauquet AT (2009). The costimulatory molecule ICOS regulates the expression of c-Maf and IL-21 in the development of follicular T helper cells and TH-17 cells. Nat Immunol.

[52] Ozaki K (2004). Regulation of B cell differentiation and plasma cell generation by IL-21, a novel inducer of Blimp-1 and Bcl-6. J Immunol.

[53] Tangye S (2013). The good, the bad and the ugly - TFH cells in human health and disease. Nat Rev Immunol.

[54] Eto D (2011). IL-21 and IL-6 are critical for different aspects of B cell immunity and redundantly induce optimal follicular helper CD4 T cell (Tfh) differentiation. PLoS One.

[55] Bai L (2017). Retrospective analysis of risk factors associated with Kawasaki disease in China. Oncotarget.

[56] Kuo H (2017). Epigenetic hypomethylation and upregulation of matrix metalloproteinase 9 in Kawasaki disease. Oncotarget.

[57] Kuo H (2016). Genome-wide association study identifies novel susceptibility genes associated with coronary artery aneurysm formation in Kawasaki disease. PLoS One.

