# Supplementary figures and images for: Variation in IL-21-secreting circulating follicular helper T cells in Kawasaki disease
Source: BMC Immunol. 2018 Dec 27;19:43. doi: 10.1186/s12865-018-0282-8 (PMC6307283; doi:10.1186/s12865-018-0282-8)

Figure S1

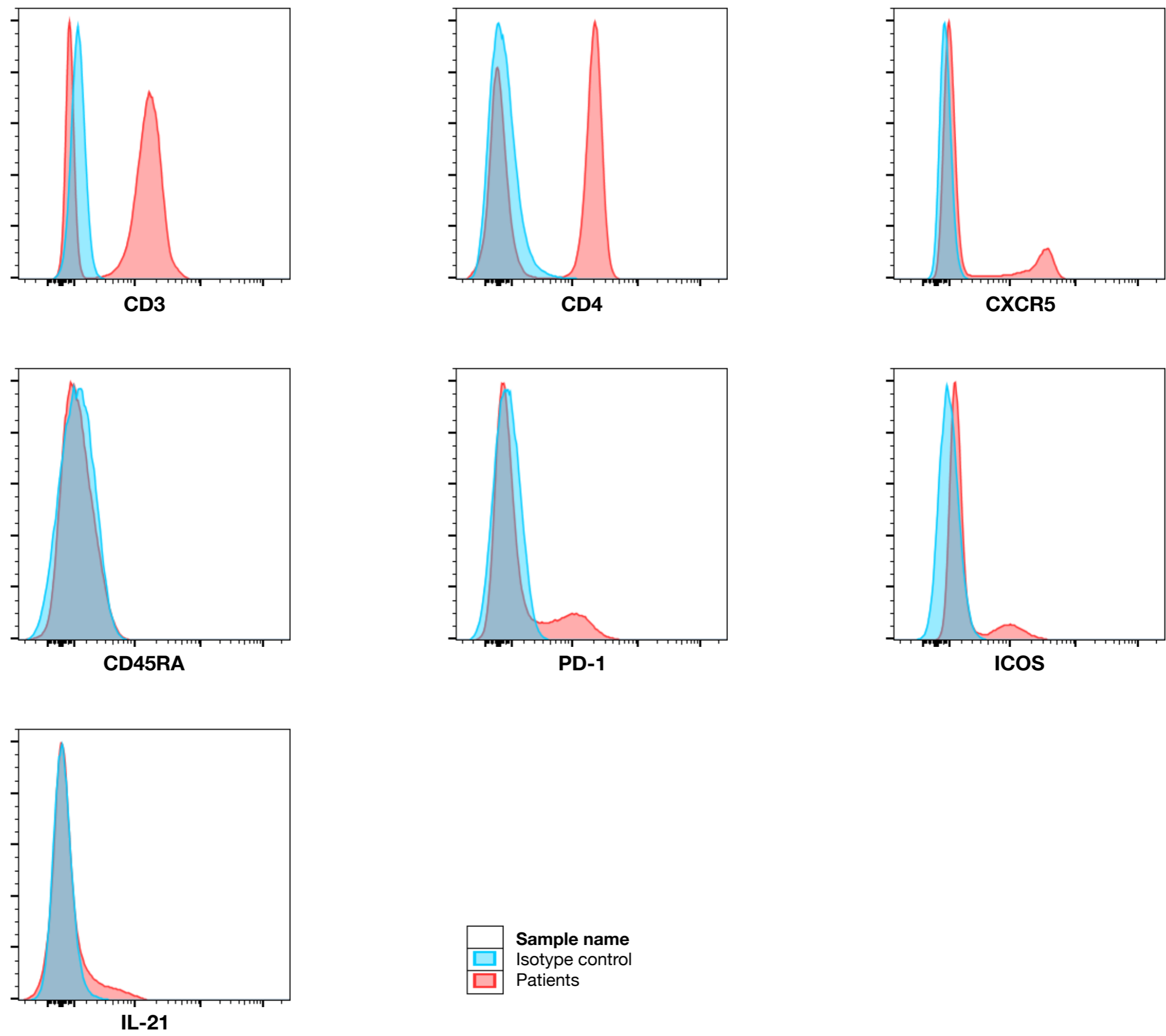

Supplement: Supplementary file 1 — Figure S1. To ensure proper gating stratery, isotype controls were used to determin the gating parameters. (PDF 104 kb) [file 12865_2018_282_MOESM1_ESM.pdf]
